# Intravitam Diagnosis of Rabies in Patients with Acute Encephalitis: A Study of Two Cases

**DOI:** 10.3390/idr14060095

**Published:** 2022-11-29

**Authors:** Monil Singhai, Vishesh Sood, Priyanka Yadav, Kiran K. Kumar, Rekha Jaiswal, Shyam Madabhushi, Pawan Dhull, Manju Bala, Sujeet K. Singh, Simmi Tiwari

**Affiliations:** 1 Centre for Arboviral and Zoonotic Diseases, National Centre for Disease Control, New Delhi 110054, India; 2 Army Hospital (R&R), Delhi 110010, India; 3 National Centre for Disease Control, New Delhi 110054, India; 4 Division of Zoonotic Diseases Program, National Centre for Disease Control, New Delhi 110054, India

**Keywords:** rabies, ELISA, CSF, Intravitam diagnosis

## Abstract

Rabies is one of the oldest known zoonotic diseases. Rhabdovirus, an RNA virus belonging to the genus Lyssavirus and family Rhabdoviridae, causes rabies. Rabies diagnosis is challenging as the rabies virus remains confined to neurons after the initial animal bite. It largely remains immune-evasive until the infection reaches the central nervous system. The bottleneck in rabies diagnosis remains the non-availability of technical expertise and failure to collect an appropriate sample. The laboratory confirmation of rabies in both antemortem and postmortem samples is important. The samples were tested for anti-rabies antibodies using quantitative ELISA. In this report, two case studies are presented to demonstrate the suitability of ELISA for the intra vitam diagnosis of rabies using cerebrospinal fluid (CSF) as a diagnostic sample. The interpretation of serology results for both vaccinated and unvaccinated individuals has been discussed in detail, which has helped to confirm the antemortem diagnosis of rabies. In this report, we observed that ELISA can be a viable alternative for anti-rabies antibody detection in CSF and can be used as a viable alternative to more technically challenging tests, such as Rapid Fluorescent Focus Inhibition Test (RFFFIT) and Immunofluorescence Assays (IFA).

## 1. Introduction

Rabies is one of the oldest known zoonotic diseases. Rhabdovirus, an RNA virus belonging to the genus Lyssavirus and family Rhabdoviridae, causes rabies with neurological manifestations. Rabies is still endemic in many parts of the world, with the maximum number of cases reported from India. Rabies cases reported from India are mainly dog-mediated with a history of an animal bite. Rabies has also been reported after being bitten by other mammals. The laboratory diagnosis of rabies is challenging. The rabies virus remains largely immune-evasive until the infection reaches the central nervous system. A clinical suspicion of rabies in humans, based on case definitions of suspected rabies cases and probable rabies cases, requires laboratory confirmation [1]. The laboratory confirmation of rabies is obtained by detecting the presence of the rabies virus antigen, viral nucleic acid, or virus-specific antibodies in an appropriate sample and at times repeat sampling is also required. The bottleneck in rabies diagnosis remains the non-availability of technical expertise and failure to collect an appropriate sample. The laboratory confirmation of rabies in both antemortem and postmortem samples is important as it helps establish rabies epidemiology, initiates public health responses such as infection control or mass vaccination of dogs [2], may prevent organ transplant-mediated rabies from donor to recipient [3,4], and initiates the palliative care of terminal cases [5]. We previously reported a case of an averted corneal transplantation in a reported case of death in a corneal donor due to acute psychosis who had a history of an animal bite five months back and turned out to be a laboratory-confirmed case of rabies [4]. There are various reports of using different diagnostic methods for the intra vitam diagnosis of rabies. However, most of the tests, including nucleic acid amplification tests (NAATs), rapid fluorescent focus inhibition test (RFFIT), direct fluorescent antibody test (FAT), and rabies tissue culture infection test (RTCIT), are not appropriate for routine diagnosis due to their requirements of expertise, dedicated laboratories or equipment, or high turnaround times. The enzyme-linked immunosorbent assay (ELISA) is a technique that has a low turnaround time and is suitable for low-resource settings. As per the antemortem diagnostic algorithm proposed by WHO [6], the anti-rabies antibody estimation in serum and CSF can be used for the laboratory confirmation of rabies.

## 2. Materials and Methods

In this report, two case studies are presented to demonstrate the suitability of ELISA for the intra vitam diagnosis of rabies using cerebrospinal fluid (CSF) as a diagnostic sample. The serum and CSF samples collected from both cases were quantitatively tested for anti-rabies antibodies using the Platelia™ Rabies II ELISA kit (BioRad, Marnes-la-Coquette, France) as per the kit instructions. For RT-PCR, the pan-lyssavirus nested RT-PCR protocol was used as per the WHO Manual on Laboratory Techniques in Rabies [7]. The FAT was performed using the anti-rabies nucleocapsid Fluoroisothiocynate Conjugate (BioRad, USA) as per the manufacturer’s instructions. For serum, the rabies vaccination status of the suspected case is required, as positive samples can be due to an immune response against the vaccine. In such a case, a paired sample demonstrating a rise in the antibody titer can be indicative of rabies. For CSF, a positive test confirms rabies; however, a negative test cannot rule out rabies, and a repeat test should be after 7–10 days post symptom onset.

## 3. Results

### 3.1. Case Study 1

A 40-year-old male with a history of a category three dog bite on 27 January 2022 on his left foot in a jungle near Babina, Uttar Pradesh, India, presented with paresthesia/tingling sensation on his left foot on 26 March 2022. The patient had not taken any local treatment/anti-rabies vaccination and did not visit any health care facility for post-exposure prophylaxis. The clinical suspicion of rabies was raised as the patient developed delirious behavior, fever, local paresthesia, hydrophobia, and aerophobia on 31 March 2022. The sequential serum and CSF samples were collected. The patient died on 18 April 2022. The results of ELISA for the serum and CSF samples of the patient are given in Table 1. The serum sample showed sufficient seroconversion after 14 days of symptom onset. As the patient was unvaccinated, the presence of rabies antibodies in the serum was considered laboratory confirmation of rabies. A paired CSF sample also showed seroconversion on the 11th day post symptom onset.

### 3.2. Case Study 2

A 38-year-old male from Datia, Madhya Pradesh, India, had a category three dog bite on his right foot on 14 December 2021 from a pet dog. The patient had taken a full course of post-exposure anti-rabies vaccination and ERIG instillation (on 15 December 2021) and anti-rabies vaccination (day 0 dose on 15 December 2021, Day 3 dose on 18 December 2021, day 7 dose on 22 December 2021, day 14 dose on 10 January 2022, and day 28 dose on 12 January 2022) as per the consultation provided by the District Hospital Datia, Madhya Pradesh, India. The patient was admitted to AIIMS, New Delhi, on 4 March 2022 with complaints of paresthesia in both legs on 28 February 2022, paralysis of legs on 1 March 2022, breathlessness on 4 March 2022, and paralysis of upper limbs on 5 March 2022. An MRI scan showed features of transverse myelitis on 5 March 2022. The patient was considered to be a suspected rabies case, and his CSF, serum, and skin biopsy samples were sent for laboratory confirmation. The results of the laboratory diagnosis of the patient are given in Table 2. The patient had sufficient anti-rabies IgG antibodies both in CSF (day 7) and serum (day 12). The serum antibody titer could not be used for the laboratory confirmation of rabies in this case, as the patient was vaccinated. However, the presence of antibodies in the CSF helped in the laboratory confirmation of the case. The RT-PCR test performed on the CSF 7 days post symptom onset was negative, and the FAT of a skin biopsy collected 9 days post symptom onset was inconclusive. The patient died on the 12th day.

## 4. Discussion

A rabies virus infection starts at the bite site where the saliva of the infected animal enters the wound [7]. The rabies virus multiplies in a localized fashion in the muscle cells. The neuromuscular junctions are the first site where the rabies virus infects the axon of the motor or sensory neurons via receptor-mediated endocytosis. The infection spreads retrogradely along the axons [7]. The virus replicates and is trans-synaptically transmitted to other neurons until the virus reaches the central nervous system (CNS) [8]. In the CNS, the virus spreads centrifugally and reaches the dorsal root ganglion. At this point, the neurological symptoms start appearing in the individual. The virus replication in the dorsal root ganglion and other parts of the brain results in inflammation; this inflammation results in associated symptoms, such as pain, paresthesia, and/or pruritus [9]. The inflammation caused in the CNS ensures a humoral response, which is essential for clearing the infection by the antibody-mediated pathway [10,11]. The antibody titers in the serum also rise at this stage. Therefore, the presence of anti-rabies antibodies in the serum of unvaccinated patients or in the CSF can directly indicate rabies infection. Detecting rabies-neutralizing antibodies in CSF or serum using RFFIT has been proposed as a viable antemortem diagnostic method for rabies [12]. Also, literature shows that ELISA gives comparable results to RFFIT [13] and the indirect fluorescence antibody (IFA) assay [14] for measuring anti-rabies antibodies. Both serum and CSF can be used for antemortem diagnosis. However, the sensitivity and specificity of antibody estimation are high for CSF [14]. In this report, we observed that ELISA is a viable alternative for anti-rabies antibody detection in CSF and has been used as a viable alternative to more technically challenging tests, such as RFFFIT and IFA for the first time in our center. Also, the two cases reported here represent the two arms of the WHO’s antemortem rabies diagnosis algorithm [5], which explains the interpretation of serology results for both vaccinated and unvaccinated individuals. Antibodies appear in CSF after the onset of neurological symptoms of rabies. It is crucial that optimum day/repeat sampling be required to demonstrate antibodies in CSF. Therefore, demonstrating antibodies in CSF by ELISA may help in the laboratory confirmation of rabies in resource-limited settings.

## Figures and Tables

**Table 1:** Quantitative anti-rabies ELISA for sequential serum and CSF samples collected from a 40-year-old male with clinical suspicion of rabies.

Sample	Days Post Symptom Onset	ELISA Result ^1^	Conc. (EU/mL)
Serum	1	No Seroconversion	-
Serum	14	Sufficient Seroconversion	3.57
Serum	16	Sufficient Seroconversion	3.91
CSF	1	No Seroconversion	-
CSF	4	No Seroconversion	-
CSF	11	Sufficient Seroconversion	0.97

^1^ Titer of <0.5 EU/mL is considered “No Seroconversion”, and titer between 0.5 EU/mL and 4 EU/mL is considered “Sufficient Seroconversion”.

**Table 2:** Laboratory tests performed for samples collected from a 37-year-old male with clinical suspicion of rabies.

Sample	Days Post Symptom Onset	Test ^1^	Results
CSF	7	ELISA (IgG)	Sufficient Seroconversion ^2^ (2.36 EU/mL)
CSF	7	RT-PCR	Negative
Skin biopsy	9	FAT	Inconclusive
Serum	12	ELISA (IgG)	High Seroconversion ^2^ (4.22 EU/mL)

^1^ ELISA (IgG)—anti-rabies IgG Enzyme-Linked Immunosorbent Assay; RT-PCR—Reverse Transcriptase Polymerase Chain Reaction; FAT—Direct Fluorescent Antibody Test. ^2^ Range 0.5 EU/mL–4 EU/mL is considered “Sufficient Seroconversion”, and a titer of >4EU/mL is considered “High Seroconversion”.

## Data Availability

Not applicable.
